# PEGASO e-Diary: User Engagement and Dietary Behavior Change of a Mobile Food Record for Adolescents

**DOI:** 10.3389/fnut.2022.727480

**Published:** 2022-03-17

**Authors:** Maurizio Caon, Federica Prinelli, Leonardo Angelini, Stefano Carrino, Elena Mugellini, Silvia Orte, José C. E. Serrano, Sarah Atkinson, Anne Martin, Fulvio Adorni

**Affiliations:** ^1^School of Management, University of Applied Sciences and Arts Western Switzerland (HES-SO), Fribourg, Switzerland; ^2^Institute of Biomedical Technologies, National Research Council, Segrate, Italy; ^3^College of Engineering, University of Applied Sciences and Arts Western Switzerland (HES-SO), Fribourg, Switzerland; ^4^Haute Ecole Arc Ingénierie, University of Applied Sciences and Arts Western Switzerland (HES-SO), St. Imier, Switzerland; ^5^eHealth Unit, Center Tecnològic de Catalunya (Eurecat), Barcelona, Spain; ^6^Experimental Medicine, Universitat de Lleida, Lleida, Spain; ^7^Human Factors Research Group, University of Nottingham, Nottingham, United Kingdom; ^8^United Kingdom Medical Research Council/Chief Scientist Office Social and Public Health Sciences Unit, University of Glasgow, Glasgow, United Kingdom

**Keywords:** mobile health technologies, mobile food record, diet monitoring, dietary target behaviors, user engagement, healthy eating habits promotion

## Abstract

**Background:**

Obesity amongst children and adolescents is becoming a major health problem globally and mobile food records can play a crucial role in promoting healthy dietary habits.

**Objective:**

To describe the methodology for the implementation of the e-Diary mobile food record, to assess its capability in promoting healthy eating habits, to evaluate the factors associated with its usage and engagement.

**Methods:**

This is a descriptive study that compared the characteristics of participants engaged in the e-Diary, which was part of the PEGASO project in which an app to provide proactive health promotion was given to 365 students at 4 European sites enrolled during October to December 2016: England (UK), Scotland (UK), Lombardy (Italy), and Catalonia (Spain). The e-Diary tracked the users' dietary habits in terms of food groups, dietary indexes, and 6 dietary target behaviors relating to consumption of: fruit; vegetable; breakfast; sugar-sweetened beverages; fast-food; and snacks. The e-Diary provided also personalized suggestions for the next meal and gamification.

**Results:**

The e-Diary was used for 6 months by 357 adolescents (53.8% females). The study showed that females used the e-Diary much more than males (aOR 3.8, 95% CI 1.6–8.8). Participants aged 14 years were more engaged in the e-Diary than older age groups (aOR 5.1, 95% CI 1.4–18.8) as were those with a very good/excellent self-perceived health status compared to their peers with fair/poor health perception (aOR 4.2, 95% CI 1.3–13.3). Compared to the intervention sites, those living in Catalonia (aOR 13.2 95% CI 2.5–68.8) were more engaged. In terms of behavior change, a significant positive correlation between fruit (*p* < 0.0001) and vegetables (*p* = 0.0087) intake was observed in association with increased engagement in the e-Diary. Similarly, adolescents who used the app for more than 2 weeks had significantly higher odds of not skipping breakfast over the study period (aOR 2.5, 95% CI 1.0–6.3).

**Conclusions:**

The users highly engaged with the e-Diary were associated with improved dietary behaviors: increased consumption of fruit and vegetables and reduced skipping of breakfast. Although the overall usage of the e-Diary was high during the first weeks, it declined thereafter. Future applications should foster user engagement, particularly targeting adolescents at high risk.

**Clinical Trial Registration:**

https://www.clinicaltrials.gov/, identifier: NCT02930148.

## Introduction

In 2016 over 340 million children and adolescents worldwide aged 5–19 were estimated to be overweight or obese (1). Excess body weight during childhood and adolescence remains a global public health emergency with important short-term and long-term consequences in adulthood (2). In an attempt to reverse this global scenario, the member states of the World Health Organization (WHO) implemented strategies to control the progression of obesity such as the “no increase in childhood overweight by 2025” as one of the six global nutrition targets in the “Comprehensive Implementation Plan for Maternal, Infant, and Young Child Nutrition” (3).

Among the key determinants of obesity, several studies reported that unhealthy dietary habits such as skipping breakfast (4), low consumption of fruit and vegetables, and high intake of energy-dense foods, sugar-sweetened beverages (SSBs), and processed food consumption (5, 6), are associated with increased likelihood of overweight and obesity in young people. Therefore, the implementation of dietary intervention programs targeting teenagers, may be crucial to influencing healthy eating patterns (7, 8).

In recent years, the availability of mobile health technologies (m-Health) to assess lifestyles, including dietary habits, and promoting dietary behavioral changes in adolescents has been proposed (9–12). There is some evidence regarding the positive impact of dietary mobile app use in increasing consumption of fruit and vegetables (13), healthy drinking habits (14), decreasing SSBs (13, 15), and eating breakfast more frequently (16) among young people. However, only a few studies reported the feasibility and/or acceptability of the nutrition apps without reporting statistics of usage (12, 17–19). Teenagers are typically the earlier and more eager adopters of mobile technology and prefer the use of a technology-based approach rather than traditional self-reported food-based questionnaires (20, 21). Compared to “conventional” instruments (pen & paper or online) used to assess dietary habits [as the food frequency questionnaire (FFQ), food record or diary, and the 24-h dietary recall] (22), technology-based tools, e.g., mobile food diaries, have the potential to reduce researcher and participant's burden and costs, improve subject's motivation, adherence, and communication, validity and reliability of dietary assessment, automate and standardize real-time data (23–25). Indeed, the real-time recording allows early detection and resolution of specific problems or can be used to provide tailored dietary advice or educational messages. The main advantages of mobile applications are that they are designed to promote frequent engagement and feedback, being both a collection and intervention tool. Furthermore, it may be useful to remind the user to provide dietary information so that the recall bias may be reduced (26). However, the design of mobile food records is crucial, and it must consider adolescents' lifestyles in order to ensure successful adoption (27, 28).

Based on this scenario, within the European PEGASO project (29), we developed an innovative theory-based mobile food record application for smartphones able to monitor the dietary behaviors of adolescents and to provide immediate educational feedback to promote behavioral changes, using a co-design approach (30). In this paper, we aimed: (i) to describe the methodology for the implementation of the e-Diary mobile food record; (ii) to evaluate the factors associated with the e-Diary usage and engagement; (iii) to assess the e-Diary capability in promoting healthy eating habits in a sample of 357 teenagers from three European countries during a 6-month time period.

## Methods

### Study Design, Setting, and Participants

This was a descriptive study undertaken within the PEGASO Fit for Future project (https://pegasof4f.ro), which was funded by the European Commission (n. 610727) and designed to provide proactive health promotion targeting adolescents through Information and Communications Technologies (ICT) in a real-world environment. A full description of the study procedures has been described in detail elsewhere (29, 30). Briefly, twelve secondary schools from four European sites England (UK), Scotland (UK), Lombardy (Italy), and Catalonia (Spain) were chosen as study settings. The unit of intervention allocation was at the school level (England, Scotland, Catalonia) or the class level (Lombardy), based on convenience and preference from the schools, and accounting for different socio-economic and cultural backgrounds (inner-city and suburban areas). The research team asked the schools to propose participation in the study to their students, taking into account gender balance, in order to enroll a study population of 350 adolescents (100 in England, Catalonia and Lombardy, and 50 in Scotland) to be assigned to the ICT intervention. Whole classes (Lombardy) or schools (England, Scotland, and Catalonia) were allocated to the intervention or the control group.

**Figure 1:**
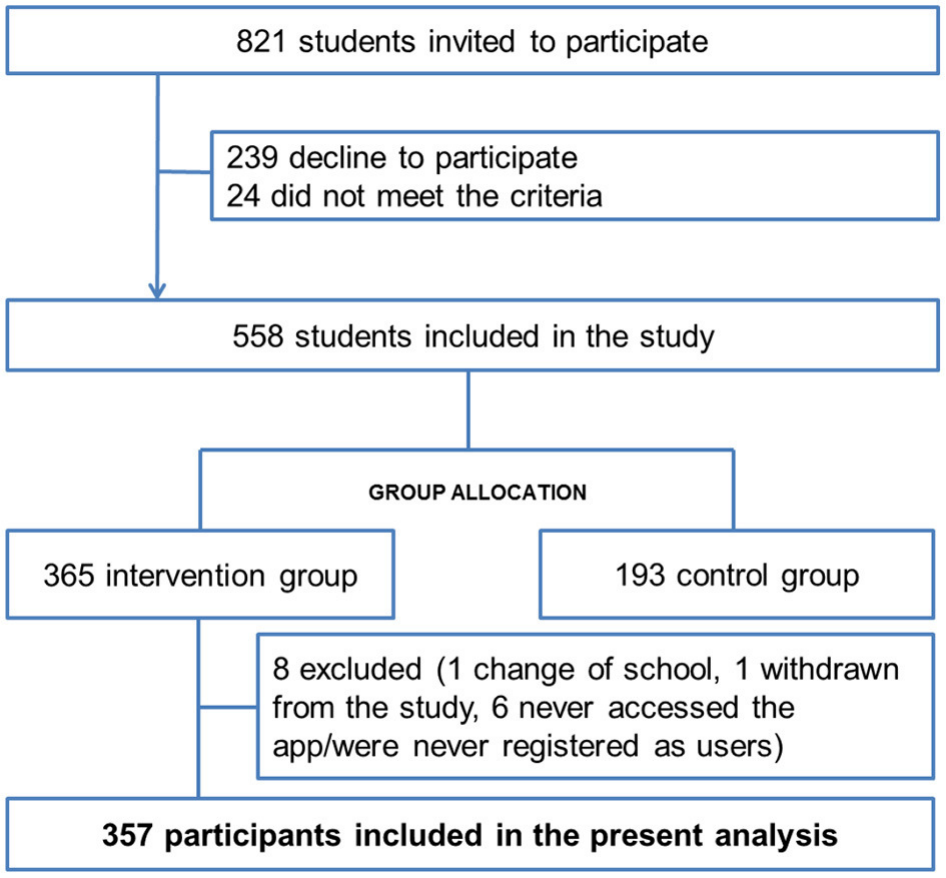
Study sample flow-chart.

Inclusion criteria were being female and male adolescents aged 13–16 years (inclusive) who agreed to participate and signed the informed consent along with their parents/guardian; had no physical or psychological condition that would prevent understanding of and participation in the intervention; were able to participate in the study for 6 months, and had adequate proficiency in the local language. The exclusion criterion was being unable to use a smartphone device or failing to meet inclusion criteria. Eight hundred and twenty-one teenagers aged 13–16 years were invited to take part in the study. Of them, 239 declined to participate and 24 did not meet the inclusion criteria, and the remaining 558 adolescents (68%, response rate) were allocated to intervention (*N* = 365) or control group (*N* = 193). Of those in the intervention group who received the PEGASO app, 8 were excluded, leaving a final sample of 357 participants for the present analysis (see Flow-chart, Figure 1). This paper focuses on the descriptive analysis of the 357 adolescents who used the mobile food record, here called e-Diary, to compare their characteristics and their engagement.

### Data Protection and Ethics

Students and their parents were provided with written study information and were allowed to ask questions before signing the consent form. They were also informed that they could withdraw from the study at any time without giving a reason and without compromising their schooling.

Data recorded by the platform or online questionnaires were stored in the Amazon Cloud in accordance with the provision of European current legislation on personal data protection: European Union General Data Protection Regulation (EU GDPR) 2016/679 (http://gdpr-info.eu/). A unique internal code for all data collected was assigned to each student to protect from unauthorized access to personal data. The study was conducted following the guidelines laid down in the Declaration of Helsinki and all procedures involving human subjects were approved by the Ethical Committee for Clinical Research (CEIC), of all four intervention sites: South East Scotland Research Ethics Committee for Scotland and England (16/SS/0163; AMO SA1), IRCCS Policlinico of Milan in Italy (212_2016) and Institut d'Investigació en Atenció Primaria de Salut Jordi Gol (IDIAP Jordi Gol) in Spain (P16/113). The study was registered at ClinicalTrials.Gov (NCT02930148).

### Procedures and Data Collection

Participating students were recruited from October 7th to December 14th, 2016 (M0) and underwent assessments at month 2 (M2), month 4 (M4), and month 6 (M6). At enrolment, an Android mobile phone already set up for the use of the PEGASO app was provided to the intervention group. Students were offered support during the study to facilitate understanding of how to use the app and to troubleshoot, any technical issues arising. Support was provided *via* direct contacts with field researchers in the schools where possible, or *via* e-mail, google hangout, FAQ site in the PEGASO web portal, video tutorial, and pdf-documentation otherwise. Mobile phones were all returned in April/May 2017 after 6 months of usage. Sociodemographic characteristics, lifestyle, anthropometric measurements, health status perception, as well as family socio-economic status were collected for all participants (both control and intervention group) using self-administered computer-based questionnaires at M0 and M6. For the present study, we considered a sub-set of variables collected at baseline. The food-based assessment was performed using the KIDMED questionnaire developed for children and adolescents and consisting of 16 items. Questions identifying negative behaviors (consumption of fast food, baked goods, sweets, and skipping breakfast) are scored with −1, while questions identifying positive behaviors (consumption of oil, fish, fruits, vegetables, cereals, nuts, pulses, pasta or rice, dairy products, and yogurt) are scored with +1. The total KIDMED score was the sum of all the items score (31). Five additional *ad-hoc* questions (Supplementary Material) were also included to detect six specific dietary habits called “Dietary Target Behaviors” (DTBs): fruit and vegetable consumption, intake of sweetened beverages, snacking habit, fast-food intake, and breakfast skipping. The selected DTBs, which are associated with overweight and obesity development at a young age based on an extensive literature search by a panel of experts, constituted the core of tailored recommendations provided by the PEGASO system (32).

The household wealth was measured using the Family Affluence Scale (FAS) (33) categorized in low, medium, and high. The adolescents' self-perceived health status (SPHS), measured using one question from the Short-form health survey (34), was classified as poor/fair, good, and very good/excellent. Adolescents' motivation of using the app was assessed with the short 4-item Perceived Competence Scale (PCS) (35) and categorized in low, medium, and high.

The participant's anthropometric measurements (height, weight and waist circumference) were measured by trained personnel using standardized procedures. Body mass index (BMI) was calculated by weight (Kg) divided by height squared (m^2^) and split in tertials as <19.7, 19.7–22.7, and >22.7. More details on the study procedures have been reported in the study protocol (29).

### e-Diary Application Development and Implementation

The e-Diary application has been developed as part of the larger PEGASO Fit for Future European project which focused on promoting healthy lifestyles to teenagers. The intervention included two wearables and an app for monitoring physical activity, a serious game with educational content, an app for exchanging earned reward points for discounts on healthy food and physical activity programs in the participants' city, and the PEGASO Companion, the main app for accessing all the aforementioned apps and services. The Companion (36, 37) integrated a virtual coach that provided the user with educational content and tried to keep the user engagement high through gamification techniques, including quizzes, challenges, badges, and points.

The capability-opportunity-motivation-behavior (COM-B) model (38) was used to identify what the adolescents needed to change for achieving the target behavior. Some behavior change techniques (BCT) were thereafter implemented throughout the Companion App to identify possible strategies to be used to facilitate change in behaviors, e.g., reminders to enter the meals in the apps and rewards for meal entries.

The e-Diary was also part of the PEGASO ecosystem and could be accessed by the user through the Companion App. The e-Diary mobile application was based on food groups rather than determining specific food intake in terms of quantity and focussed on the diversity and balance of the diet instead of specific nutrient intake based on previous literature (39). Some studies have shown associations between the excess and lack of consumption of certain food groups and body weight changes (40, 41). For this reason, strategies based on food groups may provide simple and understandable recommendations for behavioral improvements that may gain more benefit in the nutritional status than a complex modification in nutrient intake. For the e-Diary mobile application, food groups were selected based on the nutritional characteristics and similarities of foods, together with the necessity to be easily recognized and understood by the adolescents. Table 1 provides a list of the major food items classified in each food category.

**Table 1:** Description of food items included in food categories included in the e-Diary.

**Food group categories**	**List of single food items classified in the food categories**
1. Fruit (fresh)	All fresh fruit, whole, cut-up, or pureed (not canned, frozen, or dried), 100% fruit juice (not sweetened fruit juice)
2. Vegetables	All fresh and frozen (canned, or dried/dehydrated) vegetables (except potatoes beans and peas), raw or cooked, whole, cut-up, or mashed; 100% vegetable juice
3. Milk & yogurt	Whole, low-fat, and fat-free milk, all kind of yogurt (not frozen or dessert), calcium-fortified soymilk
4. Cheese & similar	Fresh soft, aged Cheese, Cream cheese (Philadelphia), cottage cheese and ricotta. Parmesan cheese
5. Bread	All kind of bread (white, whole meal, wholegrain), toasted; crackers, breadsticks, crispbreads, rice cakes
6. Breakfast cereals	Breakfast Cereals, muesli, oats, porridge, also high fiber (wholegrain)
7. Pasta/rice/potatoes	All kind of pasta, noodle, rice, barley, cous-cous, corn, rye, polenta, buckwheat, quinoa, bulgur, kamut, spelt, millet, sorghum, triticale, potatoes
8. Meat/fish/egg	Food not battered, fried or breaded. Not nuggets Beef, veal, pork, lamb, chicken, turkey, duck, hamburger and minced meat; ham, sausage, salami; white (cod, hake, trout, etc.) and blue fish (tuna, salmon, etc.), prawns, crab
9. Water	Natural or carbonated water not flavored)
10. Fast-Food menu	Burger type: hamburger sandwiches Chicken type: nuggets, fish & chips Pizza type: pizza, piadina Asian type: Chinese food, Indian food
11. Sweetened beverages	Coca cola/Pepsi/Fanta/Sprite. Sport drink; energy drink (Burn, Monster, Redbull, etc.) with sugar; tea sweetened (Estathé, Nestea, etc.). Fruit juice (with added sugar or from concentrate)
12. Snacking	Salty snack: chips, roasted nuts salted Bakery snacks: cakes, croissants, biscuits, pastries Sweet and dessert snacks: Chocolate-bar, candies, jellies, ice-cream, flan, pudding, gelatin, frozen yogurt, jam, honey roasted nuts, etc

The e-Diary was developed through a co-design process with teenagers, to take into account their preferences and their needs. Indeed, the e-Diary app was the result of an iterative participatory process, which included multiple focus groups and three cycles of co-design workshops with a total of 74 teenagers as presented in detail elsewhere (30). The e-Diary content and the food recommendations were provided by nutritionists and public health professional, and designers ensured a design with a colorful and appealing interface for teenagers, while engineers ensured a bug-free, responsive and intuitive interaction with the system. All the app content was translated into the three languages spoken by study participants, i.e., English, Catalan, and Italian.

Two types of indices were calculated based on the meal entries in the app:

1) the *dietary diversity,* which measured the degree of variation of the diet and provided immediate qualitative feedback about food groups,
2) the *dietary balance,* which measured the adequacy of intake in food group based on country-specific Food-Based Dietary Guidelines and informed on how many food servings the user has consumed.

The advantage of such indices was the ability to capture the complexity of human diet in a single value, taking into account the interactions between nutrients, food preparation methods, and eating patterns.

Besides the calculation of the dietary indices, dietary data collected with the e-Diary allowed detection of the six DTBs and to monitor behavioral changes (Table 2).

**Table 2:** Dietary target behaviors.

**Dietary target behaviors (DTBs)**	**Categories definition**	**Values**
1) Fruit consumption	≥2 servings/day; 1 serving /day; no serving/day	2; 1; 0
2) Vegetable consumption	≥2 servings/day; 1 serving /day; no serving/day	2; 1; 0
3) Sweetened beverages	Never; 1–2 times/week; 3 or more times/week	2; 1; 0
4) Snacking habit	Never; 1–2 times/week; 3 or more times/week	2; 1; 0
5) Fast-Food	Never; once/week; 2 or more times/week	2; 1; 0
6) Breakfast skipping	Eaten breakfast or not	1; 0

**Figure 2:**
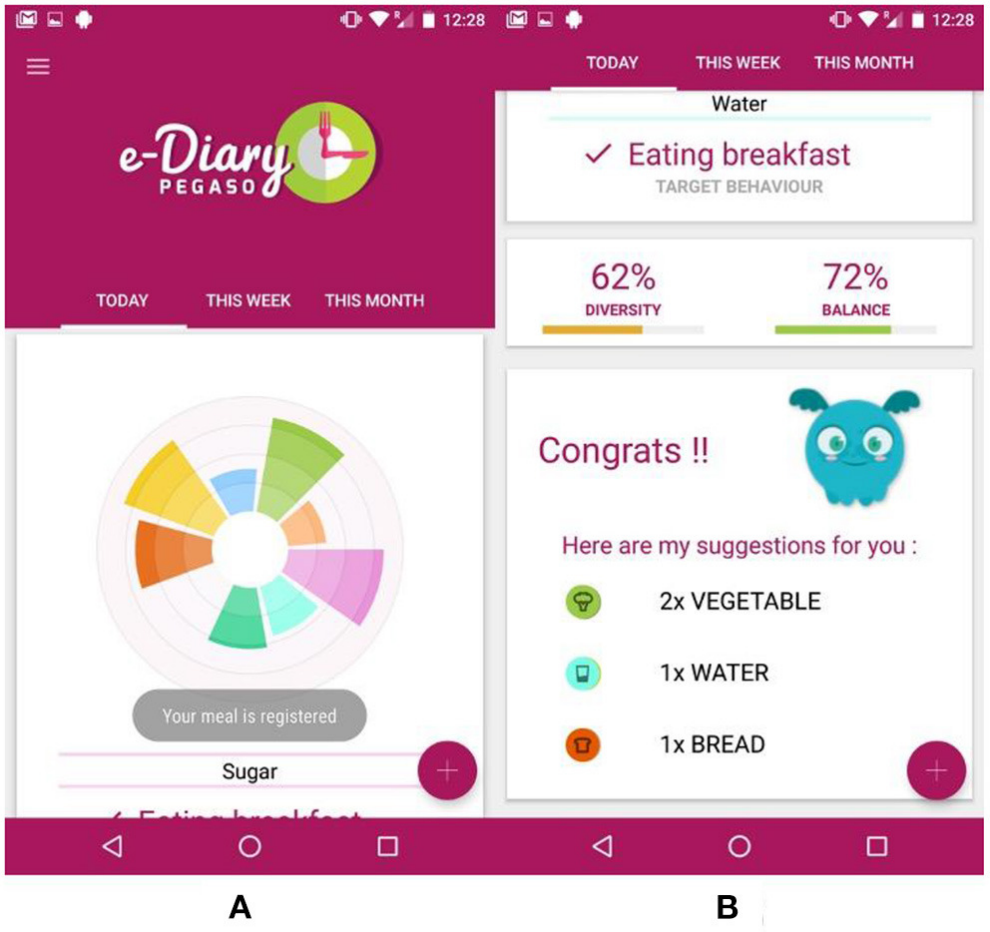
e-Diary app main interface: **(A)** the radar chart showing the daily user's meals divided in food groups; **(B)** this part of the interface shows the chosen target behavior, the diversity and balance indexes, and the recommendations for the next meal.

Figure 2 shows the main interface of the e-Diary App. The PEGASO Companion mascot welcomes the user in the e-Diary and provides tips for app first usage. A radar chart shows the user's intake for each food group as depicted in Figure 2A. Food group bars represent daily, weekly, or monthly intake in respect to recommended quantities. The most external circle corresponds to the recommendation for adolescents. Under the radar chart, the app provides a score for the diversity and the balance of the user diet (calculated on a daily, weekly, or monthly basis). Finally, the Companion mascot suggests to the user which food groups she/he can still eat to meet the daily recommendations as shown in Figure 2B. The user can tap on the plus button to enter a new meal.

**Figure 3:**
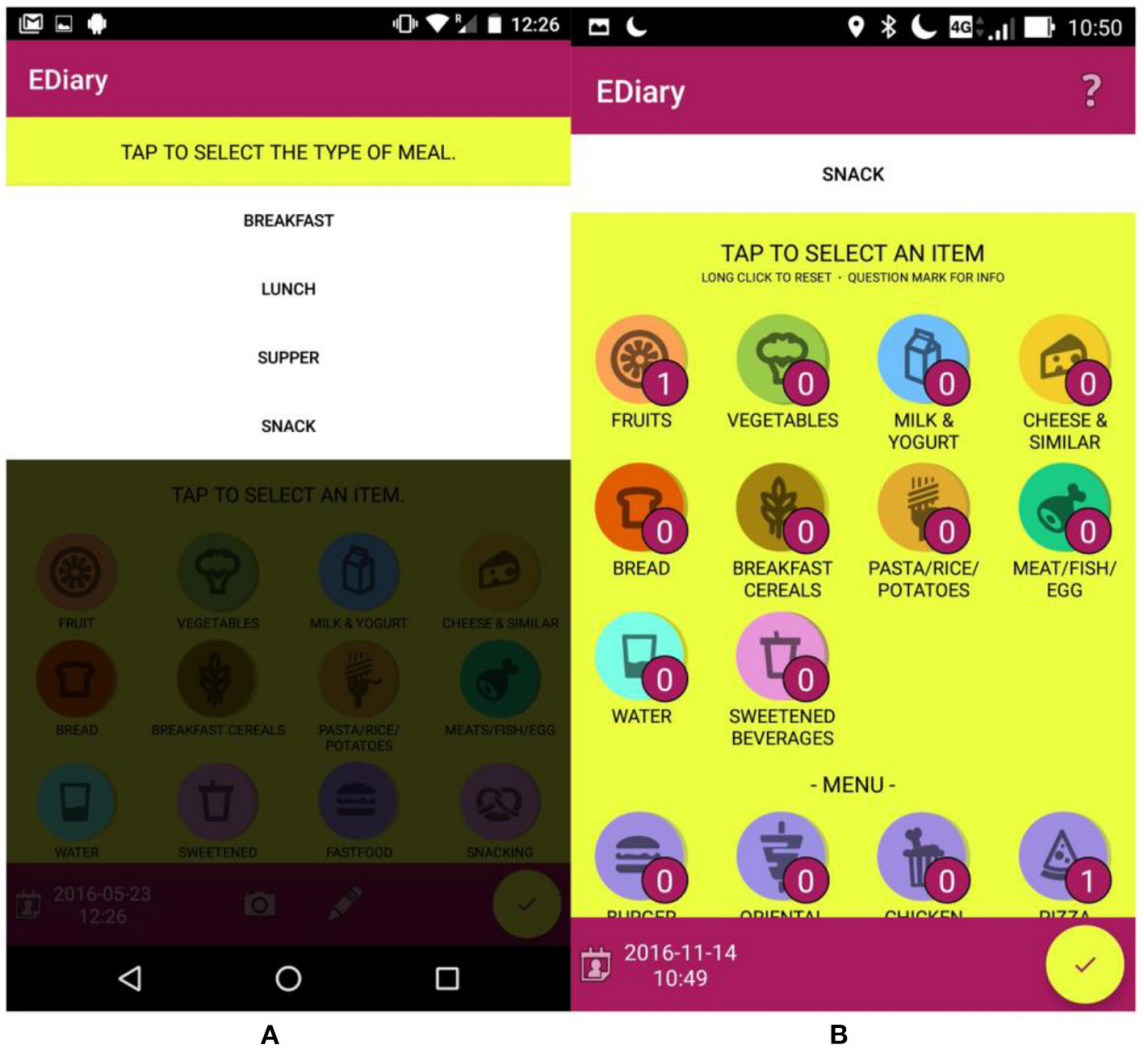
e-Diary app interface: **(A)** shows the selection of type of meal; **(B)** shows the interface for the selection of the foods eaten during the meal.

In the e-Diary interface shown in Figure 3A, the user could choose four meal types (i.e., breakfast, lunch, supper, and snack) and then the user selects the number of servings for each food category (i.e., fruit, vegetables, milk & yogurt, cheese & similar, bread, breakfast cereals, pasta/rice/potatoes, meat/fish/egg, water, sweetened beverages) as shown in Figure 3B. When participants consumed mixed dishes, they were instructed to enter each food group separately e.g. for the “pasta mixed with vegetables” dish, they entered the group “Pasta/rice/potatoes” and “vegetables” separately.

**Figure 4:**
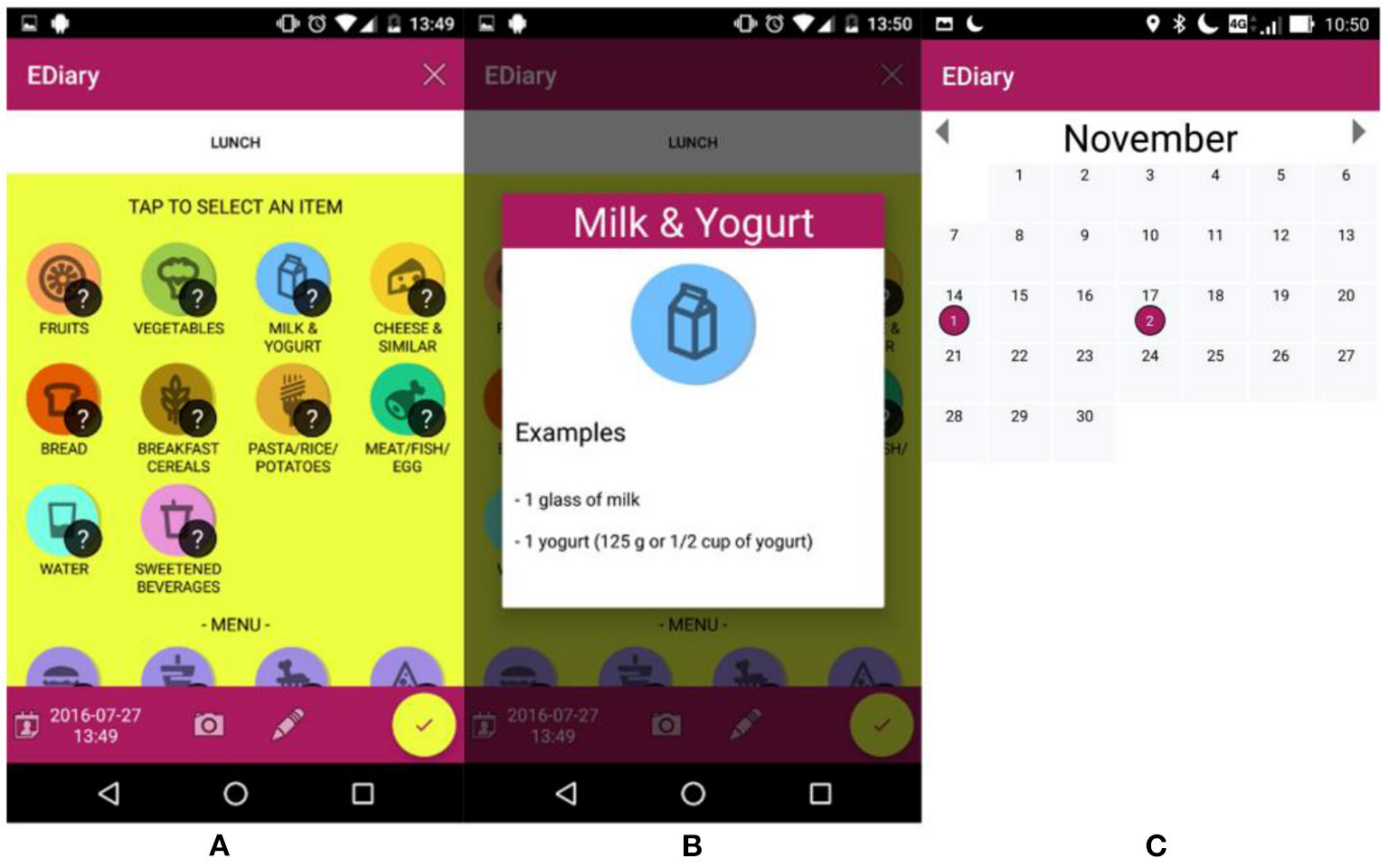
e-Diary app interface: **(A,B)** show the selection of foods with the information to guide the selection of number of servings; **(C)** shows the interface to change the information of past meals.

The choice of using food categories instead of precise food types was made to facilitate the entry process (as a result of the co-design process) while maintaining on average good validity for estimating the diversity and balance of the diet and the average intake of the main nutrient categories. Examples of food types and serving quantities were shown to the user tapping on the “?” symbol on the top-right of the application (Figures 4A,B).

To speed up the entry process, and to assess the intake of fast-food meals and snacking habits, four standard fast-food menus (burger, oriental chicken, and pizza) and three snack categories (salty, bakery, sweet & dessert) were available in the interface. Once the user confirmed the meal entry with the yellow tick button, the e-Diary app converted automatically the different food servings, menus, and snack quantities into the corresponding food nutrients and updated the radar chart in the main interface of the app, as well as the food diversity and diet balance indicators and the food recommendations. The user has the opportunity to review, modify or delete previous food entries, selecting them from a calendar interface (Figure 4C).

**Figure 5:**
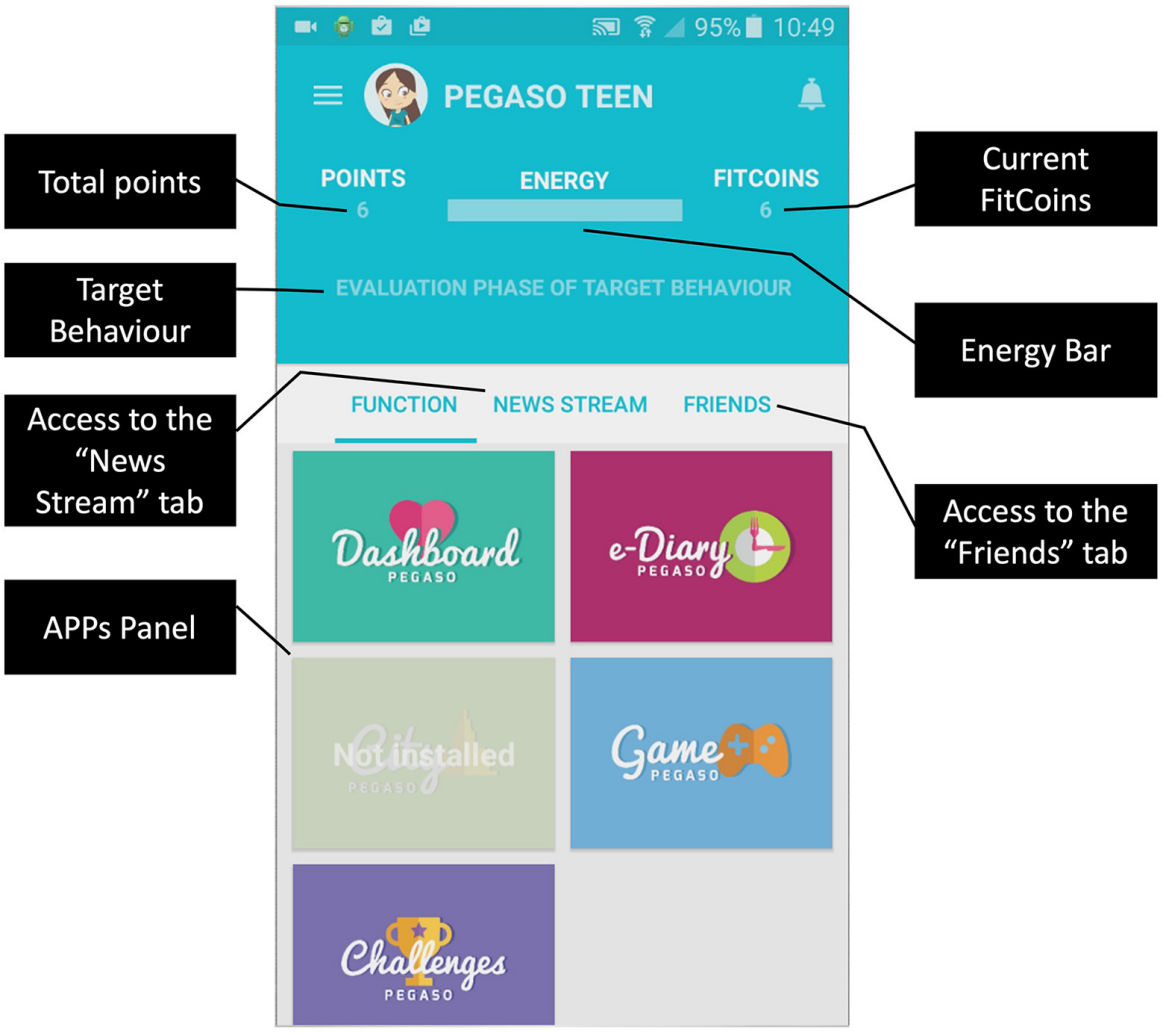
Companion app overview.

As mentioned above, the e-Diary application was accessible from the Companion app (Figure 5). However, the Companion app did not function only as a portal to access the e-Diary app but also included gamification mechanisms to motivate users to adopt the target behaviors proposed in this intervention.

**Figure 6:**
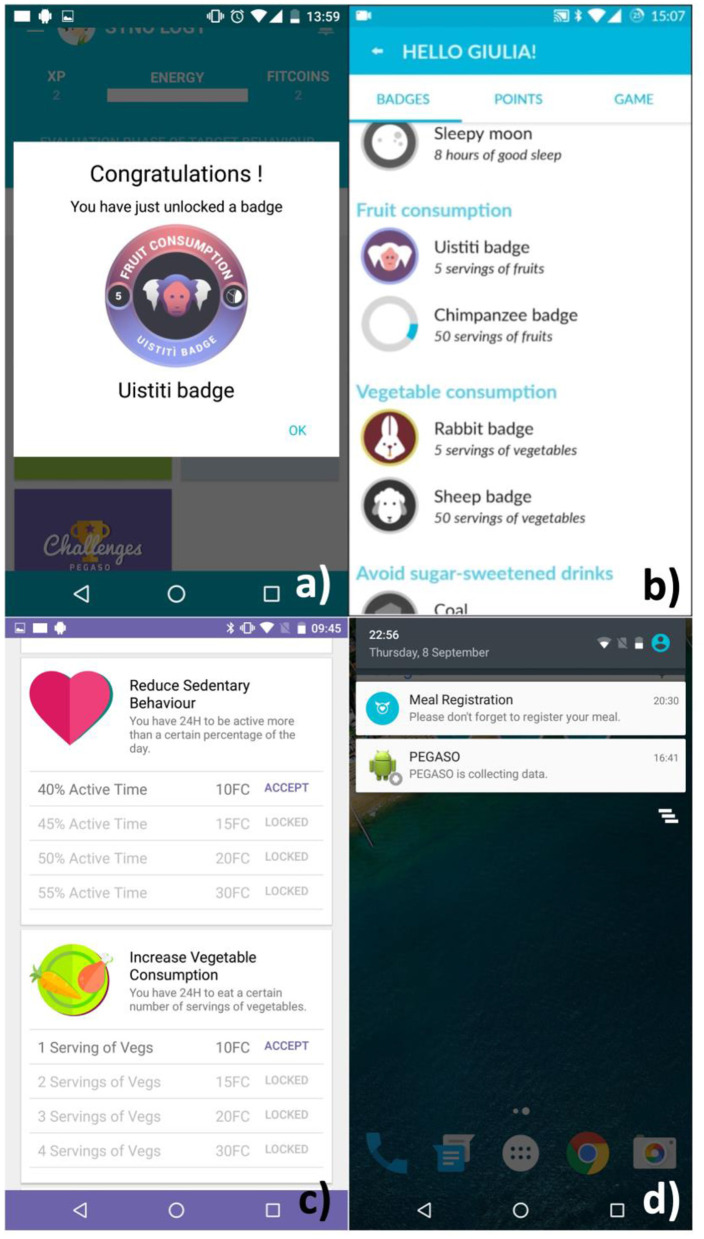
The Companion app provided: badges related to the activity on the e-Diary as depicted in **(a,b)**; challenges based on DTBs as shown in **(c)**; notifications to encourage the use of the app as in **(d)**.

In the Companion app, the user can indeed unlock badges, as depicted in Figures 6a,b or create a challenge setting a daily goal linked to the target behaviors implemented in the e-Diary (Figure 6c).

The e-Diary app sent reminders in the form of notifications once per day to encourage the users to enter their meals (Figure 6d).

The data collected (self-reported) by the e-Diary app was stored in the phone and sent daily to the cloud system for analysis of user's behaviors and adaptation of the coaching in the Companion app. The app provided some suggestions in terms of food groups based on the data filled in by the users and some messages about nutritional aspects were proposed to the teenagers by using specific questions that investigated the nutritional knowledge of the users and some notifications/tips.

### Analysis

The study used data from a variety of sources including (a) a semantic repository for storing information on food entered by the user and related indicators computed to track the user's behavior with reference to the DTBs (42), (b) Amazon Web Service Analytics for other engagement indicators (i.e., access to the app with timestamp during the study period), and (c) pen and paper food frequency questionnaire.

All of the statistical analyses were carried out using SPSS Statistics software, version 25 (IBM Corp., Armonk, NY, USA), and STATA (version 15, StataCorp LP, 347 College Station, TX, USA) software packages, and two-tailed *P*-values of 0.05 were considered statistically significant.

#### e-Diary Use and Engagement

Short-term dietary behavior was defined as the sum of the actions that PEGASO can detect during the shortest period of assessment (generally 1 day), evaluated and converted into objectively detected behaviors, which are measured according to the degree to which the user adheres recommendations. Compliance with dietary recommendations (and hence the lowest risk for obesity) received a value of 0, whilst the value 2 indicates the worst adherence to recommendations (and hence the highest risk). To capture the general trend over a longer period (1 month), long-term behavior is recognized from the sum of short-term behavior assessments. This allows the system to assess if the user has more stably changed dietary behaviors, independently from the temporary actuation of his/her habit. There are two levels of score: short-term and long-term. The objectives for each Dietary Target Behavior are decomposed in recommended actions with a related score (see Table 2). The short-term is a risk score going from 0 (compliance with recommendations) to 2 (low adherence to recommendations) for each Dietary Target Behavior computed during one specific day. The long-term score is the sum of short-term behavior assessments that captures the general trend over a longer period, further details can be found in Velickovski et al. (43).

#### Factors Associated With e-Diary Engagement

Adolescents were divided into three groups according to their engagement with the e-Diary app based on the continuous frequency of use in weeks, resulting in three categories: (i) non-users (never used), (ii) low users (used <2 weeks); and (iii) high users (used more than 2 weeks). Baseline participants' characteristics were compared using the Chi-square test according to e-Diary app usage. We estimated the odds ratios (ORs) and 95% Confidence Intervals (CIs) by using the multinomial logistic regression model to identify those baseline characteristics associated with a higher engagement in the e-Diary. Time of usage (number of weeks) grouped as never used, <2 weeks, and more than 2 weeks, as the dependent variable. The multivariate model included the following variables: sex, age, intervention sites, BMI, SPHS, FAS, and PCS. To take into account the cluster trial study design, as supplementary analysis we performed multilevel mixed-effects models considering the aforementioned seven covariates as fixed effects and countries (model A), and schools or classes nested in countries (model B) as random effects, respectively. The ORs estimated from the two models represent pooled estimation by collapsing the dependent variable from three-level to two-level. The Log-Likelihood and the Intraclass Correlation Coefficient(s) (ICC) were calculated for both models (Supplementary Table 1).

#### e-Diary Ability to Monitor Dietary Behaviors

To validate the dietary information obtained by the e-Diary, the intake of the six DTBs were compared against the dietary habits derived from the KIDMED questionnaire at baseline. The e-Diary parameters were computed from the data of the first 7 days (continuous or non-continuous) of e-Diary entries. Users, where the data range exceeded 30 days, were not included in the analysis to ensure consistency in the period between the two data sources, which resulted in 189 users for the analysis. Statistical measures of Cohen's Kappa and agreement (for the Boolean variable of breakfast skipping), and Pearson's correlation coefficients (for the Ordinal parameters) were computed. The weekly quantities for the e-Diary were estimated based on the first 7 reported days of e-Diary data.

#### e-Diary's Ability to Improve DTBs

For each time-point (M0 and M6) and for each of the six DTBs, we created 12 new variables with higher values representing higher adherence to the recommendations (see Table 2).

These new variables were obtained from the KIDMED questionnaire (Q1–Q21 in the Supplementary Material), namely for DTB-1 after combining Q1 and Q2, for DTB-2 after combining Q3 and Q4, and for TDB-3, DTB-4, DTB-5 and DTB-6 directly from Q20, Q19, Q21 and Q12, respectively. When analyzing e-Diary's ability to improve DTBs, we defined a success at M6 for each DTB as the increase in adherence to the recommendations since M0 or the maintenance of the initial condition of maximum adherence. Crude and adjusted OR with 95%CIs were estimated using binary logistic regression models to evaluate the association between the time of e-Diary usage (number of weeks) and the success in each DTB, treated as the dependent variable. In the multivariate model, we controlled for age, sex, intervention site, BMI, SPHS, FAS, PCS, and KIDMED at baseline. Additionally, we collapsed the six dependent DTB variables into a single one to analyse to joint success in three and four DTBs. Lastly, we restricted the analysis to fruit and vegetable consumption and not skipping breakfast (two out of three).

## Results

In this study, we focused on indicators associated with DTBs extracted from the use of the e-Diary which provided us with a means of monitoring dietary habits, tracking behavioral changes, determining correlations among these behaviors, and exploring the associations between these indicators and the e-Diary usage.

### e-Diary Use and Engagement

**Figure 7:**
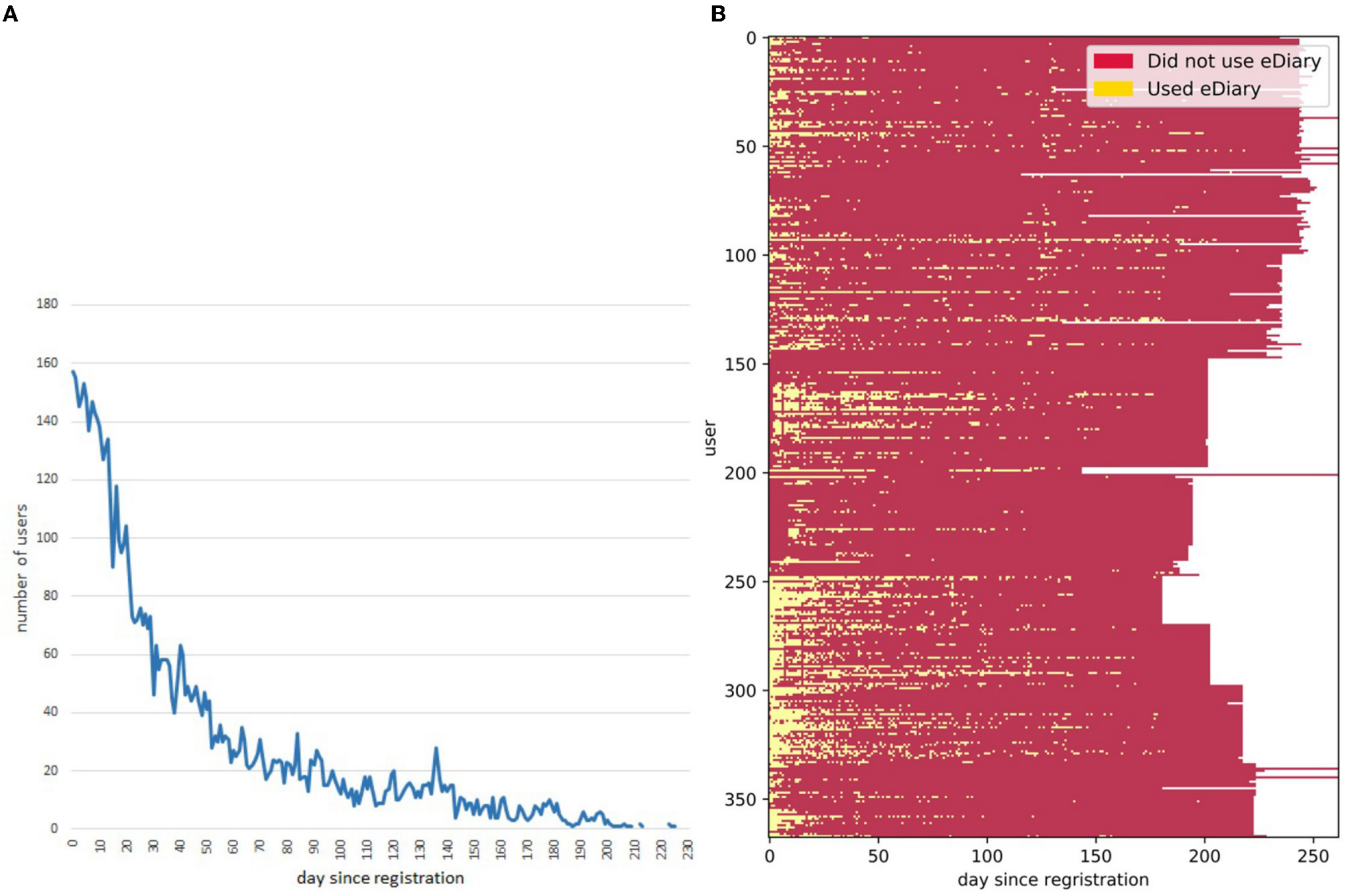
Daily usage of the e-Diary mobile app over the intervention period. **(A)** Number of users since the first access to the system. The x-axis marking time in days of the graphs have been normalized such that day 1 corresponds to the first time the user accessed the system (i.e., at registration). **(B)** Usage matrix of the total number of active e-Diary users per day. A horizontal line in the matrix represents each user. Use of the e-Diary is marked in yellow (used) or red (did not use). Shorter usage lines are due to a shorter intervention period for a group of users.

Figure 7 shows the usage of the e-Diary mobile app during the intervention period. One hundred and sixty users started to use the e-Diary immediately after the registration, which represents 45% of the studied population. The number of users declined rapidly following the first 10 days of the registration until 90 days where it was stabilized to 20 users (Figure 7A). Few users showed a continuous use of the e-Diary as shown in Figure 7B-yellow dots.

### Factors Associated With e-Diary Engagement

Table 3 summarizes the main descriptive characteristics of 357 participants with available data on the e-Diary according to the time of usage. Eighty-five percentage of the adolescents were aged 14 years or older (range 13–16), 53.8% were females, and 34.5% had BMI > 22.7 kg/m^2^. 45.7% of the participants used the e-Diary for <2 weeks. Compared with those who never used the e-Diary, the engagement was significantly higher in females, in users aged 14 years old, in those with the highest FAS, and from the Catalonia intervention site (all *P* < 0.05). No differences with respect to BMI, SPHS, KIDMED, and PCS were found in relation to engagement.

**Table 3:** Engagement with the e-Diary app based on baseline participants characteristics (*N* = 357).

		**Engagement with the e-Diary app**				
		**Never**	** <2 weeks**	**More than 2 weeks**	**Total**	***P-*value**
		*N* = 60 (16.8)*	*N* = 163 (45.7)*	*N* = 134 (37.5)*	*N* = 357 (100)*	
Sex	Females	22 (36.7)	93 (57.1)	77 (57.5)	192 (53.8)	0.014
	Males	38 (63.3)	70 (42.9)	57 (42.5)	165 (46.2)	
Age years	<14	11 (18.3)	28 (17.2)	11 (8.2)	50 (14.0)	<0.001
	14	13 (21.7)	61 (37.4)	75 (56.0)	149 (41.7)	
	15+	36 (60.0)	74 (45.4)	48 (35.8)	158 (44.3)	
Body mass index	<19.7	24 (40.0)	60 (36.8)	40 (29.9)	124 (34.7)	0.560
	19.7–22.7	15 (25.0)	49 (30.1)	46 (34.3)	110 (30.8)	
	>22.7	21 (35.0)	54 (33.1)	48 (35.8)	123 (34.5)	
Self-perceived health status	Fair/Poor	15 (25.0)	24 (14.7)	17 (12.7)	56 (15.7)	0.095
	Good	18 (30.0)	68 (41.7)	57 (42.5)	143 (40.1)	
	Very good/excellent	19 (31.7)	62 (38.0)	52 (38.8)	133 (37.3)	
	Not available	8 (13.3)	9 (5.5)	8 (6.0)	25 (7.0)	
FAS-Family affluence scale	Low	8 (13.3)	9 (5.5)	5 (3.7)	22 (6.2)	0.007
	Medium	17 (28.3)	48 (29.4)	40 (29.9)	105 (29.4)	
	High	26 (43.3)	97 (59.5)	84 (62.7)	207 (58.0)	
	Not available	9 (15.0)	9 (5.5)	5 (3.7)	23 (6.4)	
PCS–Motivation	Low	15 (25.0)	53 (32.5)	36 (26.9)	104 (29.1)	0.347
	Medium	20 (33.3)	49 (30.1)	46 (34.3)	115 (32.2)	
	High	17 (28.3)	52 (31.9)	45 (33.6)	114 (31.9)	
	Not available	8 (13.3)	9 (5.5)	7 (5.2)	24 (6.7)	
KIDMED score (mean ± sd)		5.1 ± 2.2	5.4 ± 2.7	5.8 ± 2.2	5.5 ± 2.5	0.175
Intervention site	Catalonia	6 (10.0)	43 (26.4)	67 (50.0)	116 (32.5)	<0.001
	Scotland	6 (10.0)	27 (16.6)	15 (11.2)	48 (13.4)	
	England	32 (53.3)	45 (27.6)	20 (14.9)	97 (27.2)	
	Lombardy	16 (26.7)	48 (29.4)	32 (23.9)	96 (26.9)	

Numbers in brackets represent column percentage, while ^*^ represent row percentage.

Multinomial logistic regression (Table 4) demonstrated that females used the e-Diary more than 2 weeks), almost four times more than males (aOR 3.8, 95% CI 1.6–8.8). Participants aged 14 years old used the e-Diary more than those >14 years (aOR 5.1, 95% CI 1.4–18.8). Users with a very good/excellent self-perceived health status were more engaged than users with fair/poor health perception (aOR 4.2, 95% CI 1.3–13.3). Compared to the users from the Lombardy intervention site, those living in Catalonia (aOR 13.2 95% CI 2.5–68.8) were more engaged. On the contrary, users from the England intervention site used the e-Diary less than those from the Lombardy site (aOR 0.3 95% CI 0.1–0.9). Scottish intervention site was not associated with e-Diary engagement (aOR 1.3 95% CI 0.3–5.0). No statistically significant associations were found for BMI, FAS, KIDMED, and PCS. Results from the multilevel mixed-effects models (Supplementary Table 1) were mostly consistent with those derived from the multinomial regression model. Females, 14-years old teens, and users with a better perception of their health status were more engaged in the use of the eDiary, although the pooled OR estimates were slightly lower than those derived from the main analysis.

**Table 4:** Multinomial logistic regression model between baseline characteristics of participants and the engagement with the e-Diary app.

		**Engagement with the e-Diary app**			
		** <2 weeks (vs. “Never”)**		**More than 2 weeks (vs. “Never”)**	
		**aOR [95% CI]**	***P-*value**	**aOR [95% CI]**	***P-*value**
Sex	Males^∧^	1		1	
	Females	3.4 [1.6–7.5]	0.002	3.8 [1.6–8.8]	0.002
Age years	<14^∧^	1		1	
	14	2.0 [0.6–6.2]	0.246	5.1 [1.4–18.8]	0.015
	15+	1.2 [0.4–3.6]	0.766	2.2 [0.6–8.3]	0.237
BMI-Body mass index	<19.7^∧^	1		1	
	19.7–22.7	0.8 [0.3–2.1]	0.716	1.2 [0.5–3.2]	0.665
	>22.7	0.9 [0.4–2.2]	0.769	1.2 [0.4–3.2]	0.739
SPHS-Self-perceived health	Fair/Poor^∧^	1		1	
	Good	2.8 [1.1–7.2]	0.037	3.6 [1.2–10.5]	0.021
	Very good/Excellent	3.0 [1.1–8.4]	0.039	4.2 [1.3–13.3]	0.016
FAS-Family affluence scale	Low^∧^	1		1	
	Medium	2.8 [0.8–9.9]	0.109	3.9 [0.8–18.2]	0.083
	High	2.5 [0.7–9.4]	0.183	2.6 [0.5–13.2]	0.240
PCS-Motivation	Low^∧^	1		1	
	Medium	0.8 [0.3–1.9]	0.569	0.9 [0.3–2.3]	0.799
	High	0.7 [0.3–1.7]	0.374	0.6 [0.2–1.7]	0.360
KIDMED score	*Each unit more*	1.0 [0.9–1.2]	0.722	1.1 [0.9–1.3]	0.395
Intervention site	Lombardy^∧^	1		1	
	Catalonia	5.3 [1.0–27.0]	0.045	13.2 [2.5–68.8]	0.002
	Scotland	1.4 [0.4–4.8]	0.615	1.3 [0.3–5.0]	0.720
	England	0.5 [0.2–1.4]	0.170	0.3 [0.1–0.9]	0.028

*The number of weeks of e-Diary app use was the dependent variable. Results of baseline characteristics are reported as adjusted Odds Ratio (aOR) with 95% confidence interval (CI) in brackets. The reference category of each characteristic is marked with^∧^*.

*Never e-Diary usage as reference category*.

### e-Diary Ability to Monitor Dietary Behaviors

The system tracked and gave its users feedback on the six DTBs: (i) fruit intake; (ii) vegetable intake; (iii) breakfast skipping; (iv) intake of SSBs; (v) fast-food consumption; and (vi) snack consumption. Data reported through the e-Diary has been analyzed over the intervention period to check the fulfillment of recommendations. For fruit intake, 18% of e-Diary users met on average the recommended level of two pieces of fruit or more per day, while 29% consumed at least one piece of fruit on average. For vegetable consumption, 17% of the users met the recommended level of two or more vegetables, while 29% consumed on average at least 1 vegetable. Daily breakfasts were reported to be taking on every record by 11% of e-Diary users, while 10% never recorded breakfast consumption. SSB consumption was reported regularly (on average one or more) by 16% of the e-Diary users, while 20% reported they always met recommendation by never consuming SSBs. The majority of e-Diary users (63%) occasionally consumed SSBs. Similarly, according to the data, fast-food was regularly consumed by 11% of users, while 21% never had the consumption of fast-food in their records. Finally, a substantial number of e-Diary users (39%) reported regular snack consumption (on average 1 or more snacks per day) and 53% some consumption (<1 snack per day on average), while only 9% always met recommendations by never consuming snacks (data not shown).

Table 5 shows the percentage of response agreement and concordance between the information in the e-Diary and the dietary habits collected through the paper and pencil KIDMED questionnaire at baseline. A higher response agreement was observed for the breakfast skipping behavior with a 70.8% of agreement, while fast-food (26.9%) and snack intake (28.6%) reported the lowest agreement.

**Table 5:** Response agreement between the information entered in the e-Diary app and the responses obtained by the KIDMED questionnaire (*N* = 189/304).

**Dietary target behaviors (DTBs)**	**% Response agreement**	**Pearson's rho/** **kappa**	***P-*value**
Fruit consumption	50.6	0.42	<0.001
Vegetable consumption	42.3	0.23	0.001
Sugar-sweetened beverages (SSB)	38.6	0.46	<0.001
Snacking habit	28.6	0.18	0.013
Fast-food	26.9	0.15	0.037
Breakfast skipping	70.8	0.34	<0.001

### e-Diary App's Ability to Improve DTBs

**Figure 8:**
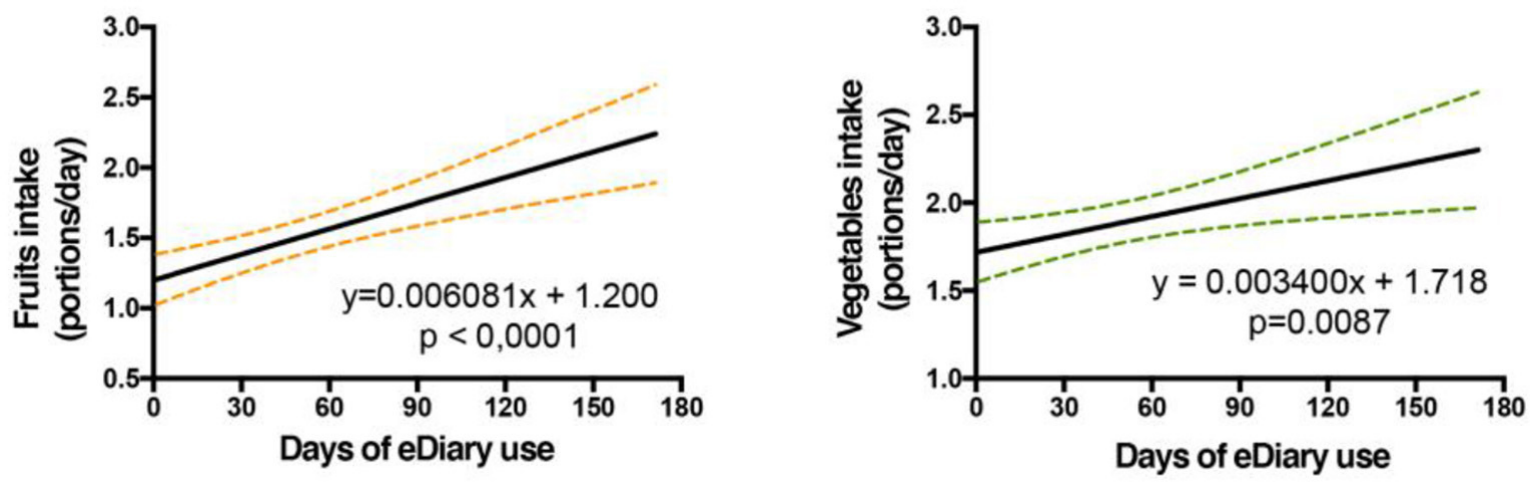
Increase in fruit and vegetable intake during the use of the e-Diary app for the users who used the app at least one time (*N* = 297): a linear regression analysis was performed between the number of servings taken per day and the days of use of the e-Diary app. Only the days that had an e-dairy record were included. *P*-values indicate the significance of the Pearson correlation between dietary behaviors and the days of e-Diary app use. The average of days of use of the eDiary is 21.7 (SD = 22.6) for the 297 participants who used the eDiary at least once.

Tracking the change in the target behaviors of each individual was not possible as entries of food records through the e-Diary app were sporadic with most users having large periods of missing records. Therefore, correlation analysis was carried out with the data as a whole (not discriminated individually) to determine if the use of the e-Diary app can be associated with the improvement of DTBs. Results from the correlation analysis are described in Table 6. There was a significant correlation between fruit (*p* < 0.0001) and vegetable (*p* = 0.0087) consumption with the use of the app (increase in fruit and vegetable intake). In this sense, since the mean intake of fruit and vegetable at the beginning of the study was 1.2 and 1.7 portions per day respectively, it would take around 296 and 382 days to fulfill the dietary recommendations (2 or more portions/per day) for each food group respectively (Figure 8).

**Table 6:** Correlation analysis between the use of the e-Diary and the change in target behaviors.

**Dietary target behaviors (DTBs)**	**Pearson coefficient**	**95% CIs**	***P-*value**
Fruit consumption	0.128	0.071 to 0.183	<0.001
Vegetable consumption	0.076	0.019 to 0.133	0.009
Sugar-sweetened beverages (SSB)	0.053	−0.004 to 0.110	0.069
Snacking habit	−0.012	−0.068 to 0.045	0.691
Fast-food	0.028	−0.029 to 0.085	0.332
Breakfast skipping	−0.027	−0.084 to 0.030	0.358

No significant correlation was observed between the e-Diary app usage and the other DTBs, suggesting that the app did not have any effects on SSB, snacking habit, fast-food, and breakfast consumption.

Table 7 reports the association between the e-Diary app engagement and the improvement of DTBs collected with the food-based questionnaire. Although in most of the DTBs no statistically significant associations were found, there was a general trend of improvement of DTBs with the increase of e-Diary usage. The only exception was breakfast consumption, where, compared to never e-Diary users, those who used the app more than 2 weeks had a significantly higher odds of not skipping breakfast over the study period in the multivariate analysis (aOR 2.5, 95% CI 1.0–6.3).

**Table 7:** Logistic regression model between the engagement with the e-Diary app and the DTBs.

	**Crude OR**		**Adjusted OR**	
	**Engaged in the e-Diary for**		**Engaged in the e-Diary for**	
	**<2 weeks**	**≥2 weeks**	**<2 weeks**	**≥2 weeks**
Target consumption of fruit or consumption increase	1.3 (0.7–2.6)	1.5 (0.8–2.9)	1.4 (0.6–3.0)	1.5 (0.6–3.4)
Target consumption of vegetables or consumption increase	1.6 (0.8–3.2)	1.0 (0.5–2.0)	1.3 (0.6–2.8)	0.7 (0.3–1.7)
Sugar sweetened beverages reduction or avoidance	1.4 (0.6–3.3)	1.6 (0.7–3.6)	1.1 (0.4–2.7)	1.2 (0.4–3.1)
Snacking reduction or avoidance	0.7 (0.3–1.9)	0.9 (0.3–2.3)	0.6 (0.2–1.9)	0.8 (0.2–2.7)
Fast–Food reduction or avoidance	1.5 (0.7–3.2)	1.7 (0.8–3.6)	1.3 (0.6–2.9)	1.3 (0.5–3.0)
Not skipping breakfast	1.1 (0.6–2.2)	2.1 (1.0–4.3)	1.2 (0.5–2.8)	2.5 (1.0–6.3)
At least 3 target behaviors attained or improved	1.4 (0.7–2.9)	1.8 (0.9–3.5)	1.1 (0.5–2.4)	1.2 (0.5–2.8)
At least 4 target behaviors attained or improved	2.2 (0.8–6.2)	2.0 (0.7–5.6)	1.9 (0.6–5.8)	1.9 (0.6–6.2)
Fruit, vegetables, breakfast (at least two out of three)	1.7 (0.8–3.3)	1.6 (0.8–3.1)	1.8 (0.7–4.8)	1.8 (0.6–5.2)

*The DTBs were used as the dependent variable. Results of baseline characteristics are reported as adjusted Odds Ratio (aOR) with 95% confidence interval (CI) in brackets*.

*Never e-Diary usage as reference category. °Models adjusted for sex, age, BMI, SPHS, FAS, PCS, intervention site and KIDMED*.

At baseline, using the food frequency questionnaire, we observed that 25 and 32.5% reported no consumption of fruit and vegetables, respectively; 53.4 and 41.5% consumed SSBs and snacks one-two times per week, respectively; 50% consumed fatty-food once a week and 30% skipped breakfast regularly. We found that females consumed SSBs and snacks less frequently but consumed fast-food and skipped breakfast more often than males.

## Discussion

The main aims of the study were to determine the short- and long-term engagement of the use of the e-Diary app; its feasibility as a tool to monitor targeted dietary behaviors and its capability to influence dietary behaviors in European adolescents aged 13–16. The principal findings are discussed below.

### e-Diary Usage and Engagement

Test participants were left free to decide whether or not to use the e-Diary and 83.2% of teenagers actually entered information in the app. 37.5% of the users engaged with the app for more than 2 weeks. There are few studies which have been conducted in a free-living environment without external control, supervision or input and in one study over a short period of time, less than half of the participants were following all of the instructions of a mobile food record (44).

It is well-known that one of the most challenging issues with self-tracking technologies is engagement over a long-term period. This has been observed in many studies and in particular, in research with adolescents it has been observed that they tend to lose interest in tracking apps or wearable devices after a few weeks (45). In the PEGASO study, only 20 out of 357 users kept entering their meals in the e-Diary for at least 90 days (37).

Results also showed that females used the e-Diary mobile food record 3.5 times more than males. This is in line with the results obtained in some previous investigations reporting that females spend more time on their phones and are more engaged in communication and mobile social application than males who spend more time playing games (17, 46–48). In particular, Boushey et al. (47) reported that females were more willing to take images of food than males. Although the aforementioned study is a mobile food record based on the automatic recognition of food, it requires the user to enter information through taking picture of the meal before and after consumption. The user experience changes in terms of entering food, in particular also because a growing trend of people sharing pictures of food on social media exists (49), nevertheless, the female population and in particular, the adolescents who have a good or excellent perception of their health were keen to use the mobile food records. This is well-documented in previous research indicating that female children, adolescents and young adults have healthier food preferences and choices and paid more attention to weight control involvement than males (50, 51). We also observed these trends during the first phase of the project, when multiple focus groups were conducted in all four intervention sites. During these focus groups teenage females, more frequently than males, expressed their particular interest in having an app to improve their diet since they valued more nutrition over physical activity in order to have a healthy life. In our study, we also found that users with a very good/excellent self-perceived health status used the e-Diary app five times more than users with fair/poor health perception. This can be interpreted as a matter of value rather than mere user experience. Indeed, people who understand the importance of a healthy diet are the ones to foster intrinsic motivation in using tools enabling the monitoring their habits in order to allow them to improve their nutritional behaviors. As expected, younger users were more engaged in the e-Diary app compared to older ones. A similar pattern has been already shown in both gaming and using communication, media and video apps (48).

We observed that users living in Catalonia were more engaged and users from the England intervention site used the e-Diary app less compared to Italians. No difference was detected between users from Scotland and the other three sites. Although we lack data for the explanation of the factor influencing the use of the e-Diary concerning the country, a possible reason may be due to the fact that in comparison with other EU countries, Spain was the bigger mobile internet user (90%) (52). Besides, results from a European cross-cultural analysis suggest that there can be some cultural differences regarding mobile phone technologies usage patterns across European regions. In Northern countries, mobile phone tools are more used for solitary activities while in Southern countries, they are more used for interpersonal activities and to maintain communication (53) and this may have improved the engagement among peers.

Our data also indicate that higher use of the e-Diary app was not related to BMI, family's socio-economic status and motivation. To date, none of the studies evaluating a nutrition/diet app related it to participant characteristics in general and BMI, socio-economic status and motivation in particular. Providing data from a large adolescent cohort is therefore an important and novel contribution. Future studies evaluating nutrition and dietary apps should consider assessing the user profile/determinants of app usage and verify if independent associations of BMI, socio-economic status, and motivation with the app use were identified. The lack of existing data could also be due to the lack of studies going beyond the feasibility and usability testing of nutrition apps with adolescents. A 2019 systematic review on the effectiveness on nutrition apps on health outcomes included only 3 (out of 41) studies in children/adolescents of which only one was conducted with a control group (54).

Finally, the reduction in the use of the e-Diary as time progresses can be explained by the lack of learning challenges perceived by the user after adapting their diet to the recommendations reflected in the app. In this sense, the implementation of more levels of learning is recommended, such as the proper selection of foods within the same food group to promote healthy food choices among others.

### Dietary Target Behaviors

The objective of the e-Diary app was to be an easy-to-use tool to monitor the consumption of DTBs in adolescents, as well as to derive personalized recommendations from the daily information collected. Despite the growing interest in eHealth technologies, the development and the application of a theory-driven app for promoting healthy dietary behaviors is not well-described in the literature (18, 55).

With respect to their ability to monitor dietary habits, mobile devices are associated with higher data quality in comparison with traditional recording methods reducing the respondents' burden and improving accuracy (23). The validation of the information collected by the e-Diary app was carried out by correlating the information entered in the mobile food record and that previously reported through a validated food frequency questionnaire. The response agreement between the two methods ranged from 26.9 to 70.8% compared to the dietary questionnaire. The highest agreement was found for breakfast skipping whereas the lowest agreement was found for a snack (28.6%) and fast-food intake (26.9%), which was reported to have a lower intake in the dietary questionnaire compared to the e-Diary. For example, 59 users reported never having had an intake of fast-food in the dietary questionnaire, however, the day-to-day record in the e-Diary reported that 72% of these users registered fast-food intake in the first 2 weeks of analysis. On the contrary, healthy dietary behaviors such as fruit and vegetable consumption were overestimated in the questionnaire compared to the e-Diary app. Indeed, 56% of users reported having one or more pieces of fruit per day in the questionnaire while no records of fruit intake were registered in the e-Diary. This is not surprising as the e-Diary is based on current and daily consumption registered in real-time, whereas the dietary questionnaire is used to measure the habitual dietary intake, which requires recall over a long-term period (24).

A possible explanation for these differences could be due to misunderstanding and internal errors due to interpretation of the question's meaning in both tools. In order to reduce this potential bias, a future implementation should incorporate messages providing more details about how to interpret the items and questions in both instruments.

The low agreement in snack and fast-food consumption could be attributed to the differences in understanding among the general population of what type of food items are included in the definition of a snack as well as fast-food. The e-Diary app included the possibility of including 3 types of snacks: salty, pastries and sweets, as well as 4 types of fast-food (burger, chicken, pizza, and Asian types). With which it could be considered that the information introduced in the e-Diary app could have a minor deviation in the concept of each item.

With respect to the reliability of the information collected in the e-Diary, the results obtained are similar to those reported in several epidemiological studies where, in general terms, there is reduced consumption of fruit and vegetables, and high consumption of SSBs and breakfast skipping. In terms of gender differences, we observed that female adolescents were characterized by lower intake of sugar-sweetened beverage and snacks but higher intake of fast-food and reported more frequently to skip breakfast compared to males. This finding is not completely in agreement with what is reported by other studies (56) as diet awareness behavior among male adolescents is known to be lower than females. Since 23% of male users (compared to 11.4% of female users) never entered data in the e-diary app, it is suspected that male users that were not interested in dietary habits decided to not use the e-Diary app. Thus, the information collected by male users who chose to use the app may be influenced by a higher awareness of dietary habits.

The second objective of the e-Diary app was improving the intake of DTBs, based on a trans-theoretical model in which the users have the opportunity to know their current intake and tailored recommendations are delivered to improve the user's behavior. Certain considerations must be taken into account that could affect the magnitude of the observed effect. On the one hand, the engagement of the use of e-Diary was low, which tends to reduce the dose of intervention received. Likewise, the number of users who consistently used the device was low and those who used it more were typically female with a higher health perceived status, a group considered to be at low nutritional risk; this could present limitations in the effectiveness of the observed results. Ideally, one would desire it to be used more by populations with higher nutritional risks, such as male teenagers with low family affluence. Taking into account these potential confounders, the use of the e-Diary was significantly associated with improved reported consumption of fruit and vegetables. The magnitude of the change is similar to other studies with a higher degree of intervention. For example, Nollen et al. (57) observed an increase in 1.8 portions per day in fruit and vegetable consumption through a hand-held computer program that provided fruit and vegetable education, tips to increase its consumption and fruit and vegetable recipes. The program required users to set intake goals and record their daily intake in a diary and was positively reinforced through a music download reward system.

The model employed for behavior change strategy is important to obtain significant results. A systematic review of iPhone Apps adherence to expert-recommended guidelines in pediatric populations suggested that for target behavior promotion, a more direct recommendation of the target behavior would increase the strength of target behavior promotion. For example, instead of recommending “more” fruit and vegetables, to recommend five servings per day (58). The e-Diary app recommendations were based on the current consumption reported by the user. The app determined each target behavior daily and made a recommendation to increase or reduce the intake of a particular food group to adjust the intake to the expert recommendations. Using this strategy, there was a significant reported improvement in the intake of fruit and vegetables, despite no effect on reducing consumption of SSBs, snacks, and fast-food.

Finally, it is important to note that several studies have observed that breakfast is an important indicator of a healthy lifestyle (59). Indeed, it may influence the total energy intake during the day as well as the food type selection in other mealtimes. Moreover, regular breakfast consumption is associated with a reduced risk of developing overweight and or obesity during adolescence in Europe (60). Despite the importance of daily breakfast consumption, breakfast skipping is common among many adolescents in western countries. In the present study, around 30% of the users were regular breakfast-skippers, other studies have observed that 7–32% of adolescents skip this meal or consume it irregularly (61, 62), where females were more frequently breakfast skippers compared to males (34.6 vs. 24.2%). Interestingly, our data seem to indicate that higher usage of the e-Diary app was associated with improving reported breakfast consumption which is in agreement with previous data (16). These results may have great relevance especially considering that breakfast consumption might be the main behavior through which it is possible to modify the other DTBs. The implementation of user-friendly mobile food record apps that incite breakfast eating might promote healthier eating patterns among adolescents.

### Strengths and Limitations

This study has several strengths. Firstly, the innovative e-Diary mobile food record development process was based on a strong theoretical background and behavior change techniques, along with the use of a participatory design approach. To our knowledge, this is one of the few studies that tested a mobile food record technology among adolescents over an extended period (i.e., 6 months) in a free-living environment without input or monitoring from external actors (e.g., parents, teachers, coaches et cetera). In addition, this study involved a relatively large sample size. Another strength is the adoption of standardized validated questionnaires to monitor dietary behaviors and to assess covariates fostering the comparison with other studies. Finally, a European multicentre design made comparison across countries possible. The e-Diary integrates a comprehensive set of functions designed to support users in improving their dietary behavior, which are:

- Real time feedback with reference to: (1) Food diversity and dietary balance indexes, (2) quantified intake of different food groups with reference to recommended daily quantities.
- Personalized recommendations for the next meal.
- Educational and motivational messages.
- Gamified functions such as badge collection and challenging a friend.

Limitations of this study should also be acknowledged. Although a large number of participants were active and engaged during the first period of the study the number rapidly declined over time indicating that long-term engagement, involvement and motivation are challenging and need to be improved. Furthermore, we cannot exclude that the relationship between e-Diary app engagement and fruit and vegetable consumption and breakfast could be partially explained by the fact that only the teens most interested in nutrition kept engaging with it over time. This potential source of selection bias may have distorted our results or conclusions because systematic differences may have occurred between those who participated and those who declined. All collected data, except the anthropometric measurements, were self-reported and therefore subjected to recall and social desirability bias. Although the analyses were adjusted for some potentially confounding factors, we cannot rule out residual confounding and confounding from other factors not considered.

## Conclusions

Assessing dietary intake accurately in adolescents is imperative to understand the complex interactions of sociological, psychological, and biological factors that may influence dietary intake. The e-Diary app is a user-friendly mobile application to register dietary intake based on the record of portions/serving size of food groups with the aim to improve dietary behavior based on self-assessment and decision empowerment. Although the engagement among users was declining over time, it was observed that in the same intervention group e-Diary users with high engagement were associated with an improvement of their eating habits, specifically the increase of breakfast consumption. However, as a negative aspect, the populations with higher nutritional risk did not show interest in using the application. In this sense, it is necessary to determine the best methods of motivation to involve this specific group.

## Data Availability Statement

The raw data supporting the conclusions of this article will be made available by the authors, without undue reservation.

## Ethics Statement

The study was conducted in accordance with the guidelines laid down in the Declaration of Helsinki and all procedures involving human subjects were approved by the Ethical Committee for Clinical Research (CEIC) of all four intervention sites: South East Scotland Research Ethics Committee in Scotland and England (16/SS/0163; AMO SA1), IRCCS Policlinico of Milan in Italy (212_2016) and Institut d'Investigació en Atenció Primaria de Salut Jordi Gol (IDIAP Jordi Gol) in Spain (P16/113). The adolescent participants' parents or legal guardian/next of kin provided written informed consent and participants provided assent to take part of the study. Written informed consent to participate in this study was provided by the participants' legal guardian/next of kin.

## Author Contributions

MC, FP, JS, and FA: conceptualization and writing—original draft preparation. MC, FP, and FA: methodology and formal analysis. MC, LA, SC, SO, and EM: software. FP, AM, SA, and FA: investigation. FA: data curation. All authors: writing—review and editing. All authors contributed to the article and approved the submitted version.

## Funding

This present study was funded by research grants of the European Commission under the 7th Framework Programme (Call identifier: FP7-ICT-2013-10; Project number: 610727). Funding has allowed the evaluation work package of the project to design the study, collect and analyses data and in writing the manuscript. AM was supported by the UK Medical Research Council (MC_UU_00022/1) and the Scottish Chief Scientist Office (SPHSU16).

## Conflict of Interest

The authors declare that the research was conducted in the absence of any commercial or financial relationships that could be construed as a potential conflict of interest.

## Publisher's Note

All claims expressed in this article are solely those of the authors and do not necessarily represent those of their affiliated organizations, or those of the publisher, the editors and the reviewers. Any product that may be evaluated in this article, or claim that may be made by its manufacturer, is not guaranteed or endorsed by the publisher.

## Abbreviations

WHO: World Health Organization
SSBs: Sugar-sweetened beverages
m-Health: Mobile health technologies
FFQ: Food frequency questionnaire
ICT: Information and Communication Technologies
EU GDPR: European Union General Data Protection Regulation
CEIC: Ethical Committee for Clinical Research
DTBs: Dietary target Behaviors
FAS: Family Affluence Scale
SPHS: Self-perceived health status
PCS: Perceived Competence Scale
BMI: Body Mass Index
COM-B: Capability-opportunity-motivation-behavior
BCT: Behavior change techniques
ORs: Odds ratios
CIs: Confidence Intervals.
