# Supplementary material for: PEGASO e-Diary: User Engagement and Dietary Behavior Change of a Mobile Food Record for Adolescents
Source: Front Nutr. 2022 Mar 17;9:727480. doi: 10.3389/fnut.2022.727480 (PMC8970185; doi:10.3389/fnut.2022.727480)
Supplement: Supplementary file 1 [file Table_1.DOCX]

Supplementary Material

We ran multilevel mixed-effects models in Stata using the meologit command for ordered logistic regression and the results are shown in Supplementary Table 1. We applied two hierarchical models, one for the two-levels (i.e., random effect of country at upper level) and one for the three-level (random effect of schools or classes nested in countries). In the equation for the level variable(s), we imposed the variance-covariance structure of the random effects to be exchangeable. For each of the two models, we obtained the OR estimates for the seven covariates considered for fixed effect, the Log Likelihood of the model and the Intraclass Correlation Coefficient(s) (ICC).

|  |  | **a) Mixed 2-levels** | | **b) Mixed 3-levels** |
| --- | --- | --- | --- | --- |
|  |  | | Country | School \| Country |
|  |  | | aOR [95% CI] | aOR [95% CI] |
| Sex | Males^ | | 1 | 1 |
|  | Females | | 1.8 [1.1-2.9] | 2.0 [1.2-3.2] |
| Age years | <14^ | | 1 | 1 |
|  | 14 | | 2.8 [1.4-5.6] | 3.0 [1.5-6.1] |
|  | 15+ | | 1.6 [0.8-3.4] | 1.8 [0.8-3.7] |
| BMI-Body Mass Index | <19.7^ | | 1 | 1 |
|  | 19.7-22.7 | | 1.3 [0.7-2.2] | 1.3 [0.8-2.4] |
|  | >22.7 | | 1.2 [0.7-2.1] | 1.3 [0.7-2.3] |
| SPHS- Self-perceived health | Fair/Poor^ | | 1 | 1 |
|  | Good | | 1.9 [1.0-3.6] | 1.9 [1.0-3.6] |
|  | Very good/Excellent | | 2.1 [1.1-4.2] | 2.0 [1.0-4.0] |
| FAS- Family affluence scale | Low^ | | 1 | 1 |
|  | Medium | | 2.2 [0.8-5.8] | 2.3 [0.9-6.4] |
|  | High | | 1.7 [0.6-4.8] | 1.8 [0.7-5.1] |
| PCS- Motivation | Low^ | | 1 | 1 |
|  | Medium | | 0.9 [0.5-1.6] | 0.9 [0.5-1.6] |
|  | High | | 0.8 [0.5-1.5] | 0.9 [0.5-1.6] |
| KIDMED score | *Each unit more* | | 1.0 [0.9-1.1] | 1.0 [0.9-1.1] |
| **LL (n° of covariates)** |  | | -298.72638 (8) | -295.7771 (9) |
| **ICC (Country)** |  | | 0.1281 | 0.0860 |
| **ICC (School \| Country)** |  | |  | 0.1036 |

**Supplementary Table 1.** Results of the multilevel mixed-effects analysis done using two hierarchical models, a) two-levels with random effect of country at upper level, and b) three-level with random effect of schools or classes nested in countries.
